# Supplementary material for: Identification of targetable epitope surfaces from the high resolution structure of the superantigen Staphylococcal enterotoxin L
Source: Sci Rep. 2026 Jun 9;16:17840. doi: 10.1038/s41598-026-54870-w (PMC13250165; doi:10.1038/s41598-026-54870-w)
Supplement: Supplementary file 1 — Supplementary Material 1 [file 41598_2026_54870_MOESM1_ESM.pdf]

Supplementary information to:

Identification of targetable epitope surfaces from the high resolution structure of the superantigen Staphylococcal Enterotoxin L

Ella Klamm[1], Caterina Curato[2], Katherina Siewert[2], and Stephen F. Marino[1*]

From the [1] Department of Biological Safety and the [2]Dermatotoxicology Study Centre, Department of Chemical and Product Safety of the German Federal Institute for Risk Assessment, Berlin, Germany

*For Correspondence: Stephen F. Marino, Stephen-Francis.Marino@bfr.bund.de

File contents:

Supplementary Figures:

S1: The structure of SEL at 1.3 Å
S2: Gating strategy
S3: Expression of further activation markers by CD4+ and CD8+ T Cells in the AIM assay
S4: Non-linear regression curves of SEL and SEB antigen-specific T cell responses
S5: Group V SE sequence comparisons with SEL

Supplementary Tables:

Table S1: Data collection and refinement statistics (molecular replacement)
Table S2: Non-linear regression model of SEL and SEB-induced T cell responses

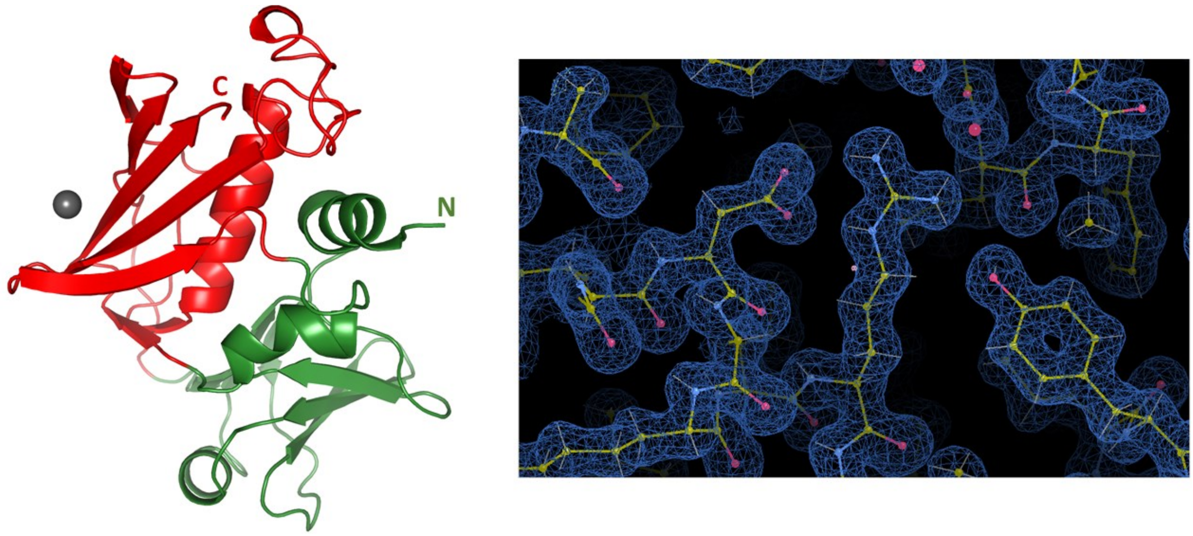

Fig S1. The structure of SEL at 1.3 Å. The left panel shows SEL rendered as ribbons with the N and C termini explicitly marked (generated using PyMOL). The N-terminal β-barrel domain is colored green and the C-terminal β-grasp domain red; the TCR binding site is on the right side of the molecule, the MHC II binding site is on the left side, at the position where a bound Zn^{2+} cation is depicted as a grey sphere. The right panel shows representative electron density (2Fo-Fc) contoured at 1.0 δ (generated using COOT).

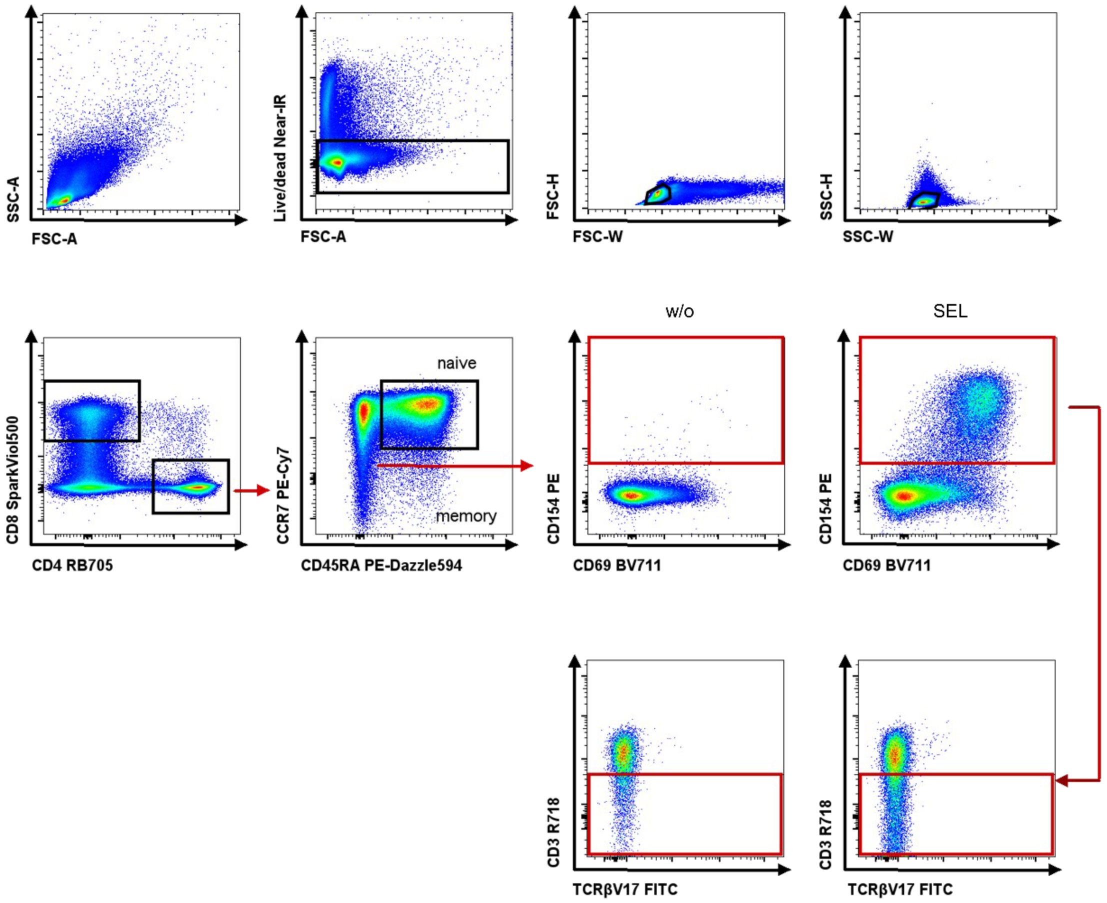

Fig S2. Gating strategy. PBMCs were stained with up to 13 fluorochrome-labelled antibodies and a dead cell stain. Sequential gating was performed on live and single cells, followed by gating on CD4+ or CD8+ memory T cells (non-naïve, naïve defined as CD45RA+CCR7+). Expression of activation markers including CD154, CD69 or CD137 was assessed. For CD154+ cells, further analysis assessed down-regulation of CD3 and the presence of Vb 17 (TRVB19) among activated T cells. Dot plots from a representative experiment with SEL [1 µg/mL].

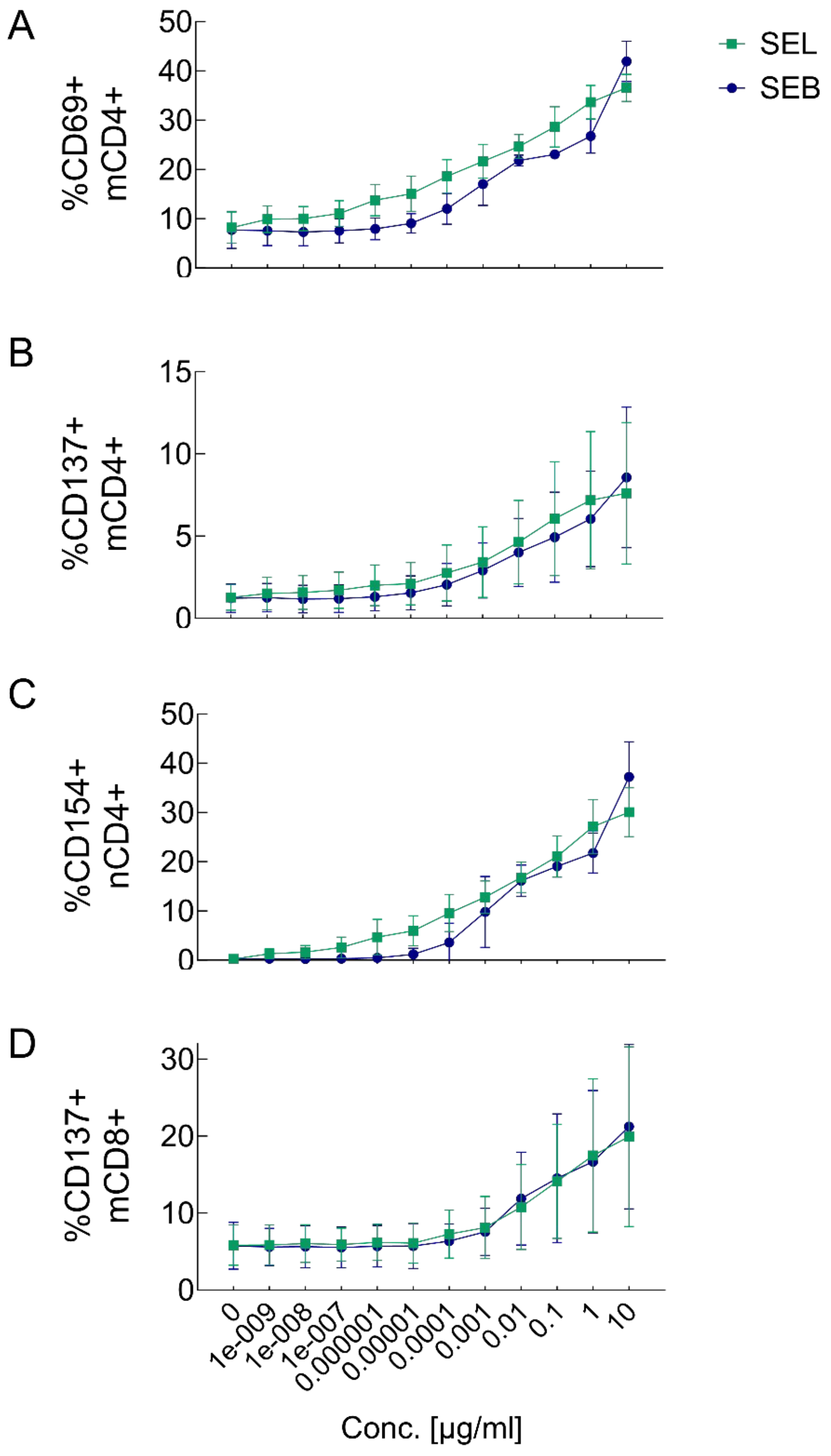

Fig S3. Expression of further activation markers by CD4+ and CD8+ T Cells in the AIM assay. PBMCs were incubated with the indicated SEL and SEB concentrations for 5 hours and analyzed by flow cytometry for upregulation of surface markers. (A) CD69 and (B) CD137 on CD4+ memory T cells, (C) CD154 on CD4+ naive T cells and (D) CD137 on CD8+ memory T cells. Lines indicate the means +/-SD. N = 4 independent experiments.

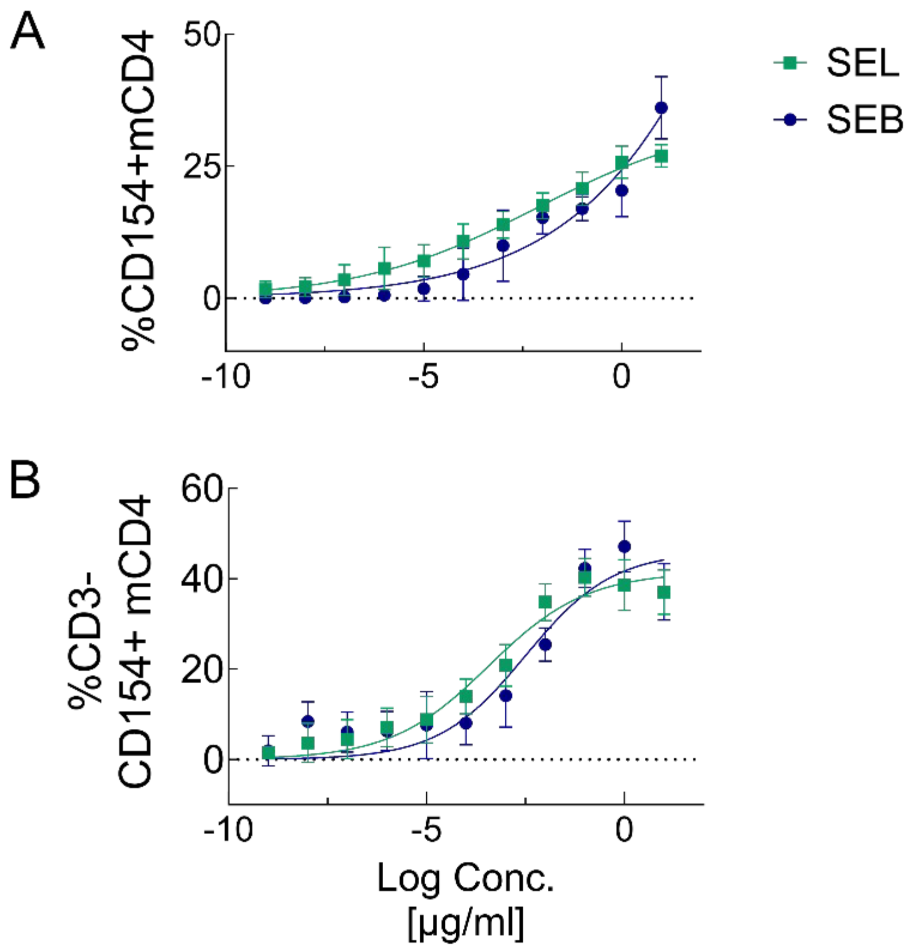

Fig S4. Non-linear regression curves of SEL- and SEB-specific T cell responses. Depicted are non-linear regression curves for the extrapolation of EC50 values and confidence intervals of SEL- and SEB-specific T cell responses (see also Table S2). Frequencies of (A) CD154+mCD4+ T cells and (B) the subpopulation that down-regulated the T cell lineage marker CD3 were used to fit the regression model after subtraction of background T cell activation without superantigen stimulation. N = 4 independent experiments.

```
SEL      NGDVGPGNLRNFYTK YEYVNLKNVKDKNSPESHRLEYSYKNDTLYAEFDNEYITSDLKGK   60
SEK      QGDIGIDNLRNFYTKKDFVDLKDVKDNDTPIANQLQFSNESYDL ISESKDFNKFSNFKGK   60

SEL      NVDVFGISYKYGSNSRTIYGGVTKAENNKLDSPRIIPINLIINGKHQTVTTKSVSTDKKM   120
SEK      KLDVFGISYNGQCNTKYIYGGVTA-TNEYLDKSRNIPINIWINGNHKTISTNKVSTNKKF   119

SEL      VTAQEIDVKLRKYLQDEFNIYGHNDTGKGKEYGTSSKFYSGFDKGSVVFHMNDGSNFSYD   180
SEK      VTAQEIDVKLRKYLQEEYNIYGHNGTKKGEEYGHKSKFYSGFNIGKVTFHLNNNDTFSYD   179

SEL      LFYTG-YGLPESFLKIYKDNKTVDSTQFHLDVEISKR---      216
SEK      LFYTGDDGLPKSFLKIYEDNKTVESEKFHLDVDISYKETI      219

>>>

SEL      NGDVGPGNLRNFYTKYEYVNLKNVKDKNSPESHRLEYSYKNDTLYAEFDNEYITSDLKGK 60
SEM      --DVGVLNLRNYYGSYPIESHQNINPDNNSLSHQLVFSKDNSTVTAEFKNVEDVKKFKNR 58

SEL      NVDVFGISYK-YGSNSRTIYGGVTKAENNKLDSPRIIPINLIINGKHQTVTTKSVSTDKK 119
SEM      AVDVYGLSYSGYCLKNKYMYGGVTLA-GDYLEKSRCIPINLWVNGNHKTISTDKVSTNKK 117

SEL      MVTAQEIDVKLRKYLQDEFNIYGHNDTGKGKEYGTSSKFYSGFDKGSVVFHMNDGSNFSY 179
SEM      IVTAQEIDTKLRRYLQEEYNIYGFNDTNKGRNYGTKSKFFSGFNTGKISFHLNDGTSFSY 177

SEL      DLFYTGYGLPESFLKIYKDNKTVDSTQFHLDVEISKR---      216
SEM      DLFDTGTGQAESFLKIYNDNKTVETDKFHLDVEISYKDES      217

>>>

SEL      NGDVGPGNLRNFYTKYEYVNLKNVKDKNSPESHRLEYSYKNDTLYAEFDNEYITSDLKGK 60
SEQ      --DVGVINLRNFYANYEPEKLQGVSSGNFSTSHQLEYIDGKYTLYSQFHNEYEAKRLKDH 58

SEL      NVDVFGISYKYGSNSRTIYGGVTKAENNKLDSPRIIPINLIINGKHQTVTTKSVSTDKKM 120
SEQ      KVDIFGISYSGLCNTKYMYGGITLA-NQNLDKPRNIPINLWVNGKQNTISTDKVSTQKKE 117

SEL      VTAQEIDVKLRKYLQDEFNIYGHNDTGKGKEYGTSSKFYSGFDKGSVVFHMNDGSNFSYD 180
SEQ      VTAQEIDIKLRKYLQNEYNIYGFNKTKKGQEYGYQSKFNSGFNKGKITFHLNNEPSFTYD 177

SEL      LFYTGYGLPESFLKIYKDNKTVDSTQFHLDVEISKR---       216
SEQ      LFYTGTGQAESFLKIYDDNKTIDTENFHLDVEISYEKTE       216

>>>

SEL      NGDVGPGNLRNFYTKYEYVNLKNVKDKNSPESHRLEYSYKNDTLYAEFDNEYITSDLKGK 60
SEI      QGDIGVGNLRNFYTKYDYIDLKGVTDKNLPIANQLEFSTGTNDLISESNNWDEISKFKGK 60

SEL      NVDVFGISYKYGSNSRTIYGGVTKAENNKLDSPRIIPINLIINGKHQTVTTKSVSTDKKM 120
SEI      KLDIFGIDYNGPCKSKYMYGGVTLS-GQYLNSARKIPINLWVNGKHKTISTDKIATNKKL 119
```

```
SEL       VTAQEIDVKLRKYLQDEFNIYGHNDTGKGKEYGTSSKFYSGFDKGSVVFHMNDGSNFSYD 180
SEI       VTAQEIDVKLRRYLQEEYNIYGHNNTGKGKEYGYKSKFYSGFNKGKVLFHLNDEKSFSYD 179


SEL       LFYTGYGLPESFLKIYKDNKTVDSTQFHLDVEISKR---       216
SEI       LFYTGDGLPVSSLKIYEDNKILQSEKFHLDVEISNVDSN       218

>>>

SEL_K     NGDVGPGNLRNFYTKYEYVNLKNVKDKNSPESHRLEYSYKNDTLYAEFDNEYITSDLKGK 60
SEL_M     NGDVGPGNLRNFYTKYEYVNLKNVKDKNSPESHRLEYSYKNDTLYAEFDNEYITSDLKGK
SEL_Q     NGDVGPGNLRNFYTKYEYVNLKNVKDKNSPESHRLEYSYKNDTLYAEFDNEYITSDLKGK
SEL_I     NGDVGPGNLRNFYTKYEYVNLKNVKDKNSPESHRLEYSYKNDTLYAEFDNEYITSDLKGK
SEL_Total NGDVDPGNLRNFYTKYEYVNLKNVKDKNSPESHRLEYSYKNDTLYAEFDNEYITSDLKGK

SEL_K     NVDVFGISYK YGSNSRTIYGGVTKAENNKLDSPRIIPINLIINGKHQTVTTKSVSTDKK
SEL_M     NVDVFGISYK-YGSNSRTIYGGVTKAENNKLDSPRIIPINLIINGKHQTVTTKSVSTDKK
SEL_Q     NVDVFGISYK YGSNSRTIYGGVTKAENNKLDSPRIIPINLIINGKHQTVTTKSVSTDKK
SEL_I     NVDVFGISYK YGSNSRTIYGGVTKAENNKLDSPRIIPINLIINGKHQTVTTKSVSTDKK
SEL_Total NVDVFGISYK YGSNSRTIYGGVTKAENNKLDSPRIIPINLIINGKHQTVTTKSVSTDKK

SEL_K     MVTAQEIDVKLRKYLQDEFNIYGHNDTGKGKEYGTSSKFYSGFDKGSVVFHMNDGSNFSY
SEL_M     MVTAQEIDVKLRKYLQDEFNIYGHNDTGKGKEYGTSSKFYSGFDKGSVVFHMNDGSNFSY
SEL_Q     MVTAQEIDVKLRKYLQDEFNIYGHNDTGKGKEYGTSSKFYSGFDKGSVVFHMNDGSNFSY
SEL_I     MVTAQEIDVKLRKYLQDEFNIYGHNDTGKGKEYGTSSKFYSGFDKGSVVFHMNDGSNFSY
SEL_Total MVTAQEIDVKLRKYLQDEFNIYGHNDTGKGKEYGTSSKFYSGFDKGSVVFHMNDGSNFSY

SEL_K     DLFYTG-YGLPESFLKIYKDNKTVDSTQFHLDVEISKR---
SEL_M     DLFYTG YGLPESFLKIYKDNKTVDSTQFHLDVEISKR---
SEL_Q     DLFYTG YGLPESFLKIYKDNKTVDSTQFHLDVEISKR---
SEL_I     DLFYTG YGLPESFLKIYKDNKTVDSTQFHLDVEISKR---
SEL_Total DLFYTG YGLPESFLKIYKDNKTVDSTQFHLDVEISKR
```

Fig S5. Group V SE sequence comparisons with SEL. Individual comparisons of each Group V SE sequence with that of SEL. The comparison sequences are in black with the corresponding color coded residues of the SEL sequence shown above them. The “Total” or consensus color coded sequence is shown last.

Table S1. Data collection and refinement statistics (molecular replacement)

	SEL
**Data collection**	
Space group	P 1 21 1
Cell dimensions	
a, *b*, *c* (Å)	32.3 , 63.86 , 48.62
α, β, γ (°)	90, 92.33, 90
Resolution (Å)	1.31 (1.31 – 1.39) *
$CC_{1/2}$	0.993 (0.228)
I / σI	6.61 (1.63)
Completeness (%)	87.48 (47.01)
Redundancy	3.23 (2.68)
**Refinement**	
Resolution (Å)	1.31 - 28.8
No. reflections	41477 (2772)
R_{work} / R_{free}	0.1233 / 0.1596
No. atoms	
Protein	1739
Ligand/ion	1
Water	153
B-factors ($Å^2$)	
Protein	21.90
Ligand/ion	13.99
Water	37.60
R.m.s. deviations	
Bond lengths (Å)	0.007
Bond angles (°)	0.94
Ramachandran outliers	0
Ramachandran favored	97.2%
Rotamer outliers	0
C-beta outliers	0

*The complete dataset was measured from a single crystal; values in parentheses are for highest-resolution shell.

Table S2. **Non-linear regression model of SEL and SEB-induced T cell responses**. Frequencies of CD154+CD4+ or CD3-CD154+CD4+ memory T cells after subtraction of background T cell activation without antigen stimulation (w/o) were fitted in a non-linear regression model (R^2>0.90). The table summarizes EC50 values and confidence intervals (CI). N = 4 independent experiments.

log(agonist) vs. response -- Variable slope (four parameters)	**CD154+CD4+** (background corrected)		**CD3-CD154+CD4+** (background corrected)	
	**SEL**	**SEB**	**SEL**	**SEB**
**Best-fit values**				
Bottom	0	0	0	0
Top	34.16	228.1	41.47	46.01
LogEC50	-2.154	5.234	-3.426	-2.495
HillSlope	0.1932	0.1763	0.3519	0.3947
**EC50**	**0.007013**	---[¥]	**0.0003754**	**0.003197**
Span	34.16	228.1	41.47	46.01
**95% CI** (profile likelihood)				
Top	30.70 to 39.51	---[¥]	36.11 to 50.75	35.23 to 271.2
LogEC50	-2.736 to -1.325	-1.220 to ---[¥]	-4.065 to -2.514	-3.623 to 6.746
HillSlope	0.1675 to 0.2212	0.1300 to 0.3239	0.2169 to 0.5929	0.1124 to 1.325
EC50 [µg/ml]	0.001836 to 0.04727	0.06028 to ---[¥]	8.612e-005 to 0.003064	0.0002382 to 5575613
**Goodness of Fit**				
Degrees of Freedom	8	8	8	8
**R squared**	**0.9973**	**0.9657**	**0.97**	**0.9033**
Sum of Squares	2.337	45.86	73.21	261.6
Sy.x	0.5405	2.394	3.025	5.719
Constraints				
Bottom	Bottom = 0	Bottom = 0	Bottom = 0	Bottom = 0
Number of points				
# of X values	11	11	11	11
# Y values analyzed	11	11	11	11

[¥] The curve of CD154+CD4+ memory T cells upon SEB stimulation did not reach saturation and therefore EC50 and CI could not be calculated by the regression model.
